# Ameliorating Effects of the Hydrogel–Stem Cell–Melatonin Combination with or Without a Mesh to Treat Experimentally Induced Liver Degeneration in Rats

**DOI:** 10.3390/life16050807

**Published:** 2026-05-12

**Authors:** Gokcen Ozgun, Deniz Yucel, Gozde Ervin Kole, Samed Ozer, Fatma Merve Antmen, Meltem Kolgazi, Nurdan Tozun, Serap Arbak

**Affiliations:** 1 Department of Medical Biotechnology, Graduate School of Health Sciences, Acibadem Mehmet Ali Aydinlar University, Istanbul 34638, Türkiye; gokcen.ozgun@acibadem.edu.tr (G.O.);; 2 Department of Histology and Embryology, School of Medicine, Acibadem Mehmet Ali Aydinlar University, Istanbul 34638, Türkiye; 3 Biomaterials Application and Research Center, Acibadem Mehmet Ali Aydinlar University, Istanbul 34638, Türkiye; 4 Experimental Animal Application and Research Center, Acibadem Mehmet Ali Aydinlar University, Istanbul 34638, Türkiye; samed.ozer@acibadem.edu.tr; 5 Department of Physiology, School of Medicine, Acibadem Mehmet Ali Aydinlar University, Istanbul 34638, Türkiye; merve.antmen@acibadem.edu.tr (F.M.A.); meltem.kolgazi@acibadem.edu.tr (M.K.); 6 Department of Gastroenterology, School of Medicine, Acibadem Mehmet Ali Aydinlar University, Istanbul 34638, Türkiye; nurdan.tozun@acibadem.edu.tr

**Keywords:** liver fibrosis, Wharton’s Jelly mesenchymal stem cells, melatonin, collagen-based hydrogel, regenerative therapy, electrospun mesh, histopathology, electron microscopy

## Abstract

Objectives: This study aimed to investigate the therapeutic efficacy of a hydrogel loaded with Wharton’s Jelly mesenchymal stem cells (WJ-MSCs) and melatonin, administered to the liver either via mesh–hydrogel implantation or intraperitoneal hydrogel injection, in a thioacetamide (TAA)-induced liver fibrosis animal model. Methods: A collagen-based hydrogel containing WJ-MSCs and melatonin was prepared for injection as well as combined with electrospun mesh for implantation. Hydrogel and mesh were characterized with respect to morphology, degradation, and mechanical properties. In in vivo studies, liver fibrosis was induced in rats by intraperitoneal injection of TAA for 6 weeks. After fibrosis induction, animals received either hydrogel injection or implantation of the combined construct. After 21 days, serum and liver tissues were collected, and biochemical, histopathological, and ultrastructural analyses were performed through comparative evaluation of experimental groups. Results: SEM results demonstrated that hydrogel, with appropriate porosity, was well integrated with the mesh without any detachment. The mesh, composed of submicron-scale fibers, exhibited a Young’s modulus of 10.37 ± 2.33 MPa. The hydrogel presented a degradation profile with a 40% mass loss in 24 h, reaching approximately 50% by day 30. Biochemical results indicated significant improvement in liver regeneration with both treatment strategies, particularly with the implanted construct. Histopathological analysis revealed decreased inflammation and hepatocyte vacuolization following both treatments; however, collagen accumulation was significantly reduced in the implant group. Ultrastructural analysis showed preserved nuclear integrity and reduced endoplasmic reticulum dilation and degenerative changes in implant group. Conclusions: The combination of WJ-MSCs and melatonin-loaded hydrogel with supportive mesh particularly enhanced tissue regeneration in liver fibrosis.

## 1. Introduction

Liver diseases resulting from complications of liver cirrhosis cause approximately 1 million deaths worldwide each year, and liver cirrhosis is the eleventh most common cause of death worldwide. The number and rate of global deaths are expected to increase in the future [1]. Numerous studies have shown that various factors, such as chemical pollutants, drugs, and alcohol, cause liver damage. Fatty liver, hepatitis, fibrosis, cirrhosis, and hepatocellular carcinoma are the most commonly observed liver diseases [2]. Chronic liver diseases are generally divided into four stages according to their severity: inflammation, liver fibrosis, cirrhosis, and end-stage liver disease (ESLD) or liver cancer [1]. Based on the literature, liver transplantation is the only effective treatment for patients with end-stage liver fibrosis (cirrhosis). However, there are issues associated with liver transplantation, such as donor shortages and long waiting lists, high costs, surgical complications, risks of immune-mediated tissue rejection, and post-transplant side effects [1,3,4,5]. Therefore, alternative approaches are needed to investigate the treatments for liver fibrosis and cirrhosis using experimental animal models.

Rat models of liver cirrhosis are usually established by the administration of hepatotoxins such as carbon tetrachloride (CCl_4_) or thioacetamide (TAA) [6,7,8,9]. TAA induces liver cirrhosis in experimental animals, producing pathological characteristics similar to those observed in human cirrhosis [6]. TAA can be administered orally or intraperitoneally, and both routes induce fibrosis and cirrhosis in rats depending on the dose and duration of exposure [6,7]. Several studies have reported that TAA is highly effective in inducing cirrhosis when administered intraperitoneally for 2–3 months at doses of 150 to 250 mg/kg, as shown in [7,8,9,10].

Mesenchymal stem cells (MSCs), due to their hypoimmunogenicity, high plasticity, and ability to differentiate into different cell types, have significant potential in tissue regenerative treatment strategies such as cell therapy and tissue engineering [11,12,13]. Umbilical cord matrix-derived WJ-MSCs are advantageous because of their easy availability, suitability for allogenic and xenogenic applications, and high differentiation potential. In addition, WJ-MSCs are attracting increasing interest in cell therapy due to their hypoimmunogenic and immunomodulatory properties [12]. Taking these features into consideration, WJ-MSCs were selected as the therapeutic cell source in the present study.

Melatonin (N-acetyl-5 methoxytryptamine), a secretory product of the pineal gland, is the focus of many research areas due to its strong antioxidant activity and protective effects against oxidative stress, including scavenging free oxygen radicals and protecting cells and tissues from free radical damage [2,14]. Various studies highlighted the anti-inflammatory, antifibrotic, antiapoptotic, anti-cancer and hepatoprotective effects of the melatonin [2,11,15]. Melatonin, a powerful antioxidant with strong free radical-scavenging properties, plays a critical role in enhancing the activity of antioxidant enzymes such as catalase, glutathione reductase, superoxide dismutase, and glutathione peroxidase, thereby delaying or inhibiting cellular damage. Studies have shown that melatonin is involved in the detoxification of ROS and free-radical intermediates by exerting its antioxidant effects [16].

Advances in biomaterials have significantly expanded their applications in drug delivery and tissue engineering, enabling localized therapeutic delivery and promoting functional tissue regeneration. Synthetic or natural polymers can be used alone or in combination to enhance various biological and physicochemical properties, such as biocompatibility, biodegradability, mechanical strength, and hydrophilicity [17]. Biodegradable synthetic polymers, such as polyglycolic acid (PGA), polylactic acid (PLA), poly (D, L-lactide co-glycolide) (PLGA), and natural polymers, such as gelatin, chitosan, collagen, hyaluronic acid, and chondroitin sulfate, have been used in liver regeneration studies [18,19].

Biomaterials, in various forms including foams, fibrous meshes, films, hydrogels and specially designed 3D constructs, are widely used in the treatment of soft tissue damage, providing structural support and restoring the functional properties of damaged tissues [17]. Hydrogels, which comprise cross-linked polymer networks, possess a flexible structure that closely mimics natural tissues [20]. One of their key advantages is that cells and bioactive components can be incorporated directly into the material during the production process [21]. Collagen is considered one of the most important biomaterials for connective tissue regeneration due to its hydrophilicity, excellent biocompatibility, minimal antigenicity, flexibility, and high yet tunable biodegradability. It also provides a biomimetic environment that supports cellular behaviors such as cell adhesion, migration and proliferation [21].

Numerous studies have demonstrated the ameliorative effects of mesenchymal stem cells and/or melatonin on liver fibrosis [12,14]. The previous literature also highlights the therapeutic potential of stem cells or melatonin delivered via hydrogels or electrospun mesh structures [18,19,22]. However, an experimental approach combining stem cells and melatonin-loaded hydrogel on an electrospun mesh for the treatment of liver injury has not yet been explored. In this study, to pave the way for clinical studies, WJ-MSCs, which are suitable for allogeneic use, were selected as the cell source.

**Figure 1:**
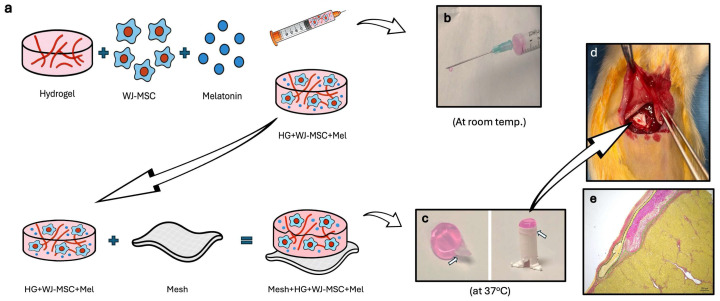
Experimental workflow illustrating the preparation of cell- and melatonin-loaded hydrogel for injection and implantation to the rat liver. (**a**) Schematic representation of the preparation of the hydrogel with WJ-MSCs and melatonin (HG + WJ-MSC + Mel) for injection and incorporation of WJ-MSCs and melatonin-loaded hydrogel with electrospun mesh for implantation. (**b**) Image of the hydrogel solution containing WJ-MSCs and melatonin in a syringe, shown in injectable form at room temperature. (**c**) The combination of WJ-MSCs- and melatonin-loaded hydrogel with electrospun mesh and its final gel state at 37 °C to form the implantation construct. White arrows indicate the electrospun mesh. (**d**) Surgical application of the implantation construct onto the surface of the rat liver. (**e**) Representative Sirius red-stained histological section of liver tissue with an implant applied to the surface. Black dashed lines indicate the mesh component, while green dashed lines indicate the hydrogel component of the implanted construct.

This study aimed to comparatively evaluate the potential regenerative effects of a hydrogel loaded with WJ-MSCs and melatonin, administered to the liver either via mesh–hydrogel implantation or intraperitoneal hydrogel injection, in a thioacetamide-induced experimental liver fibrosis animal model. For this purpose, WJ-MSCs and melatonin were incorporated into the hydrogel solution and administered to rats, with or without mesh, to assess their therapeutic efficacy in ameliorating hepatic fibrosis. In the groups without mesh, the WJ-MSCs and melatonin-loaded hydrogel solution was administered to experimental animals via intraperitoneal injection, where it was designed to undergo cross-linking and maintain gelation at 37 °C body temperature. This approach was intended to prevent leakage and dispersal of the cells and melatonin, ensuring that they remained in close proximity to the liver. In the implantation group, the WJ-MSCs and melatonin-loaded hydrogel was incorporated within the mesh and then placed directly on the liver surface. The mesh served as a structural and mechanical support for the WJ-MSC–melatonin-loaded hydrogel, thus allowing the cells and melatonin to remain on the liver surface for a longer time (Figure 1).

## 2. Materials and Methods

### 2.1. Production and Characterization of the Injection Solution and Construct for Implantation

#### 2.1.1. Preparation of the Electrospun Mesh

A solution of P(L-D, L)LA and PLGA (Corbion, Amsterdam, The Netherlands) (1:2, *w*:*w*) was prepared in chloroform:N,N-dimethylformamide (Chl:DMF; 9:1; *v*:*v*) at a concentration of 3% (*w*/*v*). Parameters, such as the distance between the syringe needle and collector, flow rate, and applied potential, were optimized. The fibers were collected on a metal plate collector under optimized conditions: 10 kV potential, 12 μL/min flow rate, and 24 cm as the distance between the tip of the needle and the collector.

#### 2.1.2. Preparation of Hydrogel for Injection and Implant Construction

A hydrogel solution was prepared using collagen type I from rat tail, 10× DMEM, 1× DMEM, dH_2_O, 1 M NaOH, and 7.5% NaHCO_3_ according to the collagen supplier’s protocol, and the final collagen concentration was 3 mg/mL in the hydrogel solution. For characterization studies, the hydrogel solution was placed into 96-well plates and kept at 37 °C for 45 min for gelation (Figure 1a). The hydrogel remained in a solution state at room temperature, allowing intraperitoneal injection near the liver; however, at body temperature (37 °C), it underwent gelation. For in vivo implantation studies, the electrospun mesh was placed on a custom-designed 3D-printed transwell, after which the hydrogel solution was added onto the mesh. Following incubation at 37 °C for 45 min, the hydrogel gelled on the mesh, and the construct was then ready for implantation onto the liver surface.

#### 2.1.3. Characterization of the Electrospun Mesh and Hydrogel

To examine the morphologies of electrospun mesh, hydrogel, and implantation construct were coated with 40 nm thick gold (Au) under vacuum with a vacuum sputter coater (Leica EM ACE200, Wetzlar, Germany) and examined with scanning electron microscopy (Thermo Fisher Scientific Quatrro S ESEM, Eindhoven, The Netherlands). Tensile mechanical test studies of the electrospun meshes of (P(L-D,L)LA-PLGA) (*n* = 5) were studied under a 1 mm/sec test speed with the universal testing machine (Shimadzu AGS-X Universal Test Machine, Kyoto, Japan). The stress–strain curves were obtained, and the Young’s moduli of the samples were determined using the slope of the stress–strain curve.

To determine the swelling degree of collagen-based hydrogels, the hydrogels (*n* = 4) were first freeze-dried. The dried samples were then immersed in PBS (0.1 mM, pH 7.4) and their weight change by water absorption was recorded. When the samples reached maximum weight, it was reported as the weight of the hydrogel in the swelling state. The swelling degree (%) was calculated as (Ws − Wd)/Wd × 100, where Ws and Wd are the weights of the hydrogel at the swelling state and dry state, respectively [23]. To determine degradation, the hydrogels (*n* = 4) were frozen at −80°C, freeze-dried overnight, and their weights were recorded as the initial weight. The freeze-dried hydrogels were then placed in PBS at 37 °C on a shaker. Hydrogel weights were measured every 15 min for the first hour and every 30 min for the following 3 h after freeze-drying.

#### 2.1.4. Culture of Human Umbilical Cord-Derived Wharton’s Jelly Mesenchymal Stem Cells

In this study, umbilical cord matrix-derived Wharton’s Jelly mesenchymal stem (WJ-MSCs) cells were kindly provided by Assoc. Prof. Deniz Yucel (Acıbadem Mehmet Ali Aydınlar University) and used as the cell source, with the approval of the Acıbadem University and Acıbadem Healthcare Institutions Medical Research Ethics Committee (ATADEK, 2023/03-91; 24 February 2023). WJ-MSCs were isolated from the human umbilical cord matrix using explant culture and stored in liquid nitrogen vapor as a cell stock [24]. The frozen cells were thawed at 37 °C in the growth medium consisting of DMEM:F12 (1:1) supplemented with 10% FBS, 100 U/mL penicillin, 100 μg/mL streptomycin, and 1 ng/mL bFGF. After centrifugation, the cell pellet was resuspended in the growth medium, transferred into a tissue culture flask, and the cells were cultured in the growth medium by changing the medium every 2 days. When the cells reached confluency, they were detached from flasks by trypsinization using 0.05% Try-EDTA for 5 min at 37 °C. The cell pellet was then resuspended in growth medium, and the cell suspension was transferred into new tissue culture flasks for subculturing. The morphology of the WJ-MSCs was examined under a light microscope (ZeissAxio Lab A., Oberkochen, Germany).

The expressions of cell surface antigens of the isolated cells were analyzed with a flow cytometer (BD Biosciences, San Jose, CA, USA). MSC markers, such as CD105, CD73, CD44, and CD90; hematopoietic lineage markers, such as CD34 and CD45; and immunogenic antigens, such as HLA-ABC and HLA-DR, were investigated. WJ-MSCs (P3), cultured in a tissue culture flask, were trypsinized and then centrifuged at 1500 rpm for 5 min. The cell pellet was resuspended in the growth medium, and the cells were transferred into tubes as 5 × 10^5^ cells/tube in 1% BSA (in PBS, 0.1 mM, pH 7.4). Samples were centrifuged at 1500 rpm for 5 min at +4 °C, and then the cells were incubated with antibodies (FITC-CD105, PerCP-CD73, PerCp-CD90, PE-CD44, FITCCD45, PE-CD34, PerCp-HLA-DR, PE-HLA-ABC, PerCp-IgG2α, FITC-IgG1, PerCp-IgG1, and PE-IgG1) for 1 h at +4 °C. After incubation, the cells were washed twice with PBS and fixed with 1% PFA for 10 min at room temperature. The cells were then centrifuged at 1500 rpm for 5 min at +4 °C, and the pellet was resuspended in PBS. The resulting cell suspension was transferred into tubes and analyzed with a flow cytometer to determine the percentage of cells expressing the indicated cell surface antigens.

To investigate the morphology and cytoskeletal organization of human WJ-MSCs, cells were stained with FITC-conjugated phalloidin and counterstained with DAPI for actin filaments and nuclei, respectively. After 80–90% confluency, cells were seeded onto fibronectin-coated glass slides at a density of 1 × 10^4^ cell/slide and cultured for 3 days in the growth medium. Then, the cells were fixed with 4% PFA at room temperature. Following this, the cells were incubated in 0.1% Triton-X-100 solution for permeability. After washing with PBS, the cells were incubated in 1% BSA blocking solution (in PBS). Afterwards, the cells were incubated in FITC-conjugated phalloidin solution (1:100) at 37 °C for actin filaments, and counterstained with 4′-6-diamidino-2-phenylindole (DAPI) at room temperature for nuclei. The cells were examined with a Laser Scanning Confocal Microscopy (LSCM, Zeiss LSM 700, Oberkochen, Germany).

#### 2.1.5. Viability of WJ-MSCs Within the Hydrogel

WJ-MSCs at a density of 1 × 10^6^ cells/hydrogel were integrated into the hydrogel solution, of which the preparation method was given in Section 2.1.2 [15]. WJ-MSCs in the hydrogel were cultured in DMEM:F12 (1:1) and supplemented with 10% FBS, 100 U/mL penicillin/100 μg/mL streptomycin for 5 days. Cell viability and morphology within the hydrogel were evaluated in vitro prior to in vivo applications.

Cell viability was investigated on the fifth day of culture by staining the cell-loaded hydrogel with the live/dead assay. The growth medium was removed from the culture, and the staining solution was directly added onto the cell-loaded hydrogels. The cells were incubated for 30 min at 37 °C, and the cells in the hydrogel were examined with LSCM. The live and dead cells were counted from the LSCM images to determine the percentage of cell viability.

#### 2.1.6. Incorporation of WJ-MSCs and Melatonin-Loaded Hydrogel with the Electrospun Meshes

A hydrogel solution containing 1 × 10^6^ WJ-MSCs/hydrogel and 1 µM melatonin/hydrogel was prepared. The implantation construct was produced by adding the hydrogel solution onto the electrospun mesh, which was then placed into the 3D-printed transwell. This set-up (Figure 1c) enabled the establishment of the mesh–hydrogel combination construct after incubation at 37 °C for 45 min to allow gelation. In in vivo applications, the hydrogel side of the construct directly faced the liver tissue, while the electrospun mesh covered the surface of the implantation site. It was anticipated that the hydrogel’s gel form would improve the adhesion of the mesh to the tissue surface.

For in vivo implantation studies, the electrospun mesh was placed into a custom-designed 3D-printed transwell, and then hydrogel solution was added onto the mesh. Following incubation at 37 °C for 45 min, the hydrogel gelled on the mesh, and the construct was then ready for implantation onto the liver surface.

### 2.2. In Vivo Studies

#### 2.2.1. Experimental Animals

Wistar albino male rats (*n* = 30), aged 6–8 weeks old (220–250 g), were used in this study. During the experiment, rats were kept in a laboratory environment at 22 ± 2 °C under a standard light/dark (12/12 h) cycle and were fed with normal pellet feed and allowed to drink water freely. This study has been approved by Acıbadem Mehmet Ali Aydınlar University Experimental Animals Local Ethics Committee (ACU-HADYEK) (Decision no: 2023/6; 22 February 2023). The study was conducted according to the criteria of international guidelines for animal research. The experimental protocol was carried out at Acıbadem Mehmet Ali Aydınlar University Experimental Animals Research Center.

#### 2.2.2. Immunohistochemical Evaluation of the Immune Response to the Xenogeneic Application of WJ-MSCs

Before starting the experimental groups, the potential immune response risk in rats following implantation of human WJ-MSCs was evaluated by intraperitoneal injection of these cells. Immunofluorescence analysis was conducted to assess whether the xenogenic application induced an immune response in rats. Rats were administered with WJ-MSCs intraperitoneally during the sixth week to mimic the treatment model. After 3 weeks, the animals were dissected, and liver tissue samples were taken and stored at −80 °C. Cryosections of the liver tissues were immunostained against CD45 surface protein to evaluate host immune response and examined with LSCM. The LSCM images were analyzed for CD45 fluorescence intensity using the ImageJ program.

#### 2.2.3. Experimental Design

Rats were randomly assigned into five experimental groups (6 rats/group): control group—rats were given the vehicle solution (saline) 3 times a week for 6 weeks; MSCs group—rats were given the vehicle solution (saline) 3 times a week, and at the end of sixth week, MSCs (10^6^ cells/rat) were injected intraperitoneally; injury group—rats were given TAA (200 mg/kg, i.p., Sigma-Aldrich Chemical Co., St. Louis, MO, USA) 3 times a week for 6 weeks to induce hepatic fibrosis; injection group—TAA (200 mg/kg, i.p., 3 times a week for 6 weeks) and hydrogel + WJ-MSCs + Mel (single i.p. administration, containing 1 × 10^6^ cells and 1 μM melatonin in a total volume of 300 μL of hydrogel) were simultaneously injected in rats; and implant group—TAA (200 mg/kg, i.p., 3 times a week for 6 weeks) and mesh + hydrogel + WJ-MSCs + Mel were implanted on the surface of the liver at the sixth week (Figure 1d).

To perform the implantation, rats were anesthetized by administering 3% isoflurane via inhalation. Then, the abdominal region was shaved and cleaned with antiseptic solution, and an approximately 2 cm wide incision was made in the midline of the abdomen at the level of the liver to expose the liver surface. After washing with DMEM F:12 solution [22], the WJ-MSC–melatonin-loaded hydrogel combined with the mesh (hybrid structure) was implanted on the liver surface.

The hydrogel consisting of type I collagen is intended to create an adhesive effect for the mesh on the liver surface. After the surgical procedure has been completed, skin and subcutaneous sutures were placed, and the rats were taken to postoperative care. For this purpose, Dichloron (0.02 mg/300 mg) and Sefazole (0.2 mg/300 g) were administered intramuscularly to the rats once a day for 3 days following the operation.

The 3-week treatment period began with the placement of the mesh on the liver surface and injection of hydrogel. At the end of week 9, all rats were sacrificed, and the blood serum samples and liver tissues were collected for biochemical, histochemical, immunohistochemical, and ultrastructural evaluations.

#### 2.2.4. Biochemical Analysis

##### Biochemical Tests for Blood Serum Samples

Prior to decapitation, blood samples were extracted from the heart for biochemical analyses and placed in dry vacuum tubes. Blood samples were centrifuged at 4000 rpm for 15 min to obtain serum. Serum samples were analyzed using total bilirubin (TB), alanine aminotransferase (ALT), aspartate aminotransferase (AST), and alkaline phosphatase (ALP) kits (OttoBC132, OttoBC127, OttoBC128, OttoBC124, Otto Scientific, Ankara, Türkiye) with a colorimetric method. The measurements were conducted on a fully automated biochemical analyzer (Mindray BS400, Mindray, Shenzhen, China).

##### Measurement of Oxidative Stress Markers in Liver Tissue Samples

Malondialdehyde (MDA) and glutathione (GSH) level measurements in liver tissue

Liver tissue samples were homogenized in a 10% trichloroacetic acid solution, followed by centrifugation. The supernatants were separated and centrifuged again. MDA levels as a marker of lipid peroxidation were determined spectrophotometrically at 535 nm absorbance using a molar extinction coefficient of 1.56 × 10^5^ M^−1^ cm^−1^. For GSH measurement, the modified Ellman’s method was used. GSH levels were determined spectrophotometrically at 412 nm.

Measurement of liver Myeloperoxidase (MPO) activity

MPO activity was determined spectrophotometrically by measuring the H_2_O_2_-dependent oxidation of o-dianisidine·2HCl. Liver tissue samples (0.2–0.5 g) were homogenized in 0.5% HETAB (50 mM potassium phosphate buffer, pH 6) and centrifuged. The supernatant was removed, and the pellet was re-homogenized in 0.5% solution in 50 mM K_2_HPO_4_. The samples were incubated with o-dianisidine·2HCl, H_2_O_2_, and the pellet. The reaction was terminated with 2% sodium azide. One unit of enzyme activity was defined as the change in absorbance at 460 nm.

Measurement of superoxide dismutase (SOD) activity

Superoxide dismutase (SOD) catalyzes the conversion of the toxic superoxide radical (O_2_•), generated during oxidative energy processes, into hydrogen peroxide and molecular oxygen. In this method, xanthine and xanthine oxidase (XOD) were used to generate superoxide radicals, which subsequently reacted with 2-(4-iodophenyl)-3-(4-nitrophenyl)-5-phenyltetrazolium chloride (INT) to form a red formazan dye. SOD activity was determined using a fully automated device, with measurements taken at an endpoint absorbance of 505 nm.

#### 2.2.5. Histopathological Analysis

##### Light Microscopical Examination

Liver tissue samples were collected from all experimental groups and fixed in 10% neutral-buffered formalin solution. The tissue samples were placed into a tissue processor and dehydrated by increasing concentration of ethanol series. Following dehydration, the tissues were cleared with xylene and then infiltrated with paraffin. After paraffin embedding, tissue blocks were cut at approximately 5 µm thickness using a rotary microtome (Thermo-Shandon™ Finesse™ ME, Waltham, MA, USA).

The Hematoxylin and Eosin (H&E) staining method was applied to sections to observe morphological changes, including vacuolization, vasocongestion, and inflammatory cell infiltration. The degree of tissue damage was scored on a scale from 0 to 3 (0: no damage, 1: mild damage, 2: moderate damage, 3: severe damage) [25]. The Sirius red staining method was used to assess the distribution and content of collagen fibers in the liver tissue. The degree of fibrosis was quantitatively evaluated using ImageJ software (version 1.44, National Institutes of Health, Bethesda, MD, USA) based on micrographs obtained by light microscopy.

##### Immunofluorescence Analysis

Cryosections of liver tissue (approximately 15 µm) were fixed with 4% formaldehyde solution (at room temperature). These slides were treated in blocking solution for 30 min and incubated with anti-galectin-3 primary antibody (Ready to use, Cedarlane Laboratories, Burlington, ON, Canada) at overnight at 4 °C. These sections were cleaned in PBS (0.1 mM, pH 7.4) and incubated with the secondary antibody (Alexa Fluor 488; 1:2000 dilution) (Cat. No: A32740, Invitrogen, Carlsbad, CA, USA) at room temperature. Liver sections were incubated with DAPI at room temperature in a dark environment and mounted with Flouromount. The sections were analyzed under LSCM. The intensity in the sections was calculated using the ImageJ software.

##### Transmission Electron Microscopical Examinations

Liver tissue samples were fixed in 2.5% 0.1 M PBS (0.1 mM, pH 7.4) glutaraldehyde solution and post-fixed in 1% osmium tetroxide. Tissue samples were dehydrated by passing through ascending concentration of ethanol series and pure propylene oxide, embedded in Epon 812, and then polymerized at 60 °C [26]. Ultrathin sections of 60 nm thickness were taken and examined under a transmission electron microscope (Thermo Fisher Scientific TALOS L 120 C, The Netherlands) [26].

### 2.3. Statistical Analysis

Statistical analysis was performed using GraphPad Prism 8.0 (GraphPad Software, San Diego, CA, USA). One-way or two-way ANOVA was used for statistical analysis, and Tukey’s test was applied for comparative analysis.

## 3. Results

### 3.1. Characterization and In Vitro Assessment of the Injectable Hydrogel and Implantation Construct

#### 3.1.1. Characterization of Electrospun Fibrous Mesh and Hydrogel

**Figure 2:**
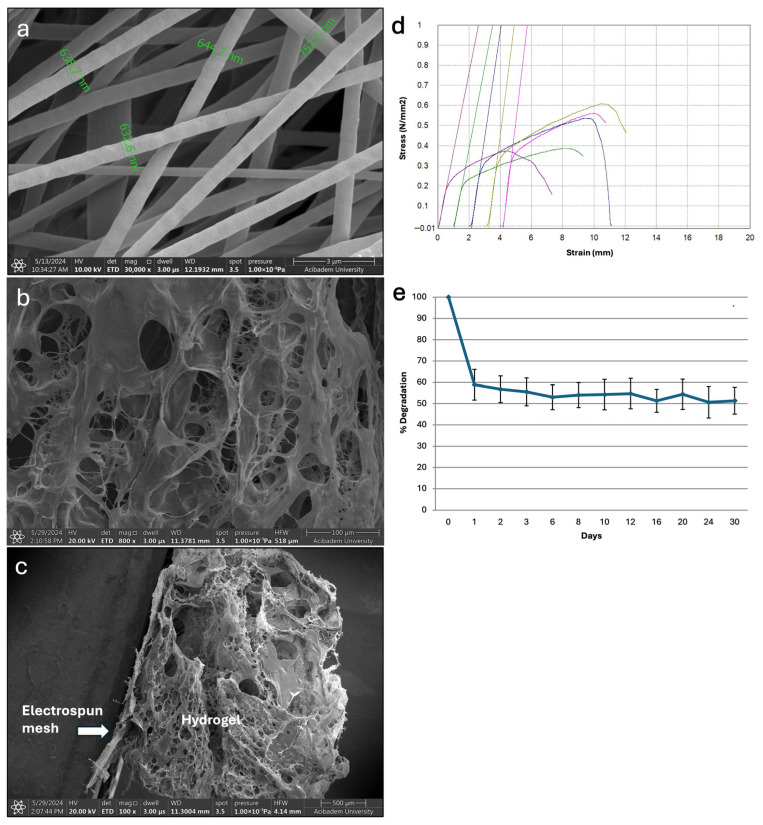
Morphological and mechanical characterization of electrospun mesh, hydrogel, and their combination. SEM images of (**a**) the electrospun mesh, (**b**) collagen-based hydrogel, and (**c**) mesh–hydrogel combination (white arrow indicates the electrospun mesh). (**d**) Stress–strain curve of electrospun mesh. (**e**) Degradation profile of the hydrogel showing percentage of weight loss over time.

The electrospun fibrous meshes were fabricated using a blend of P(L-D,L)LA and PLGA (1:2, *w*/*w*) at a polymer concentration of 3%, with an applied voltage of 10 kV, a flow rate of 12 μL/min, and a tip-to-collector distance of 24 cm. After optimization studies, uniform and bead-free fibers were obtained under these conditions (Figure 2a). The collected fibers, with diameters ranging between 500 nm and 1000 nm, formed a porous fibrous mesh structure. Gel formation of the hydrogel was confirmed within 45 min at 37 °C. SEM analysis showed that the obtained hydrogel exhibited a porous structure that allowed cell infiltration and proliferation (Figure 2b). As shown in Figure 2c, the hydrogel was well integrated with the electrospun mesh and the combined construct remained stable without detachment.

Tensile mechanical testing of the electrospun meshes revealed a Young’s modulus of 10.37 ± 2.33 MPa, indicating adequate mechanical strength for the intended purpose of this study (Figure 2d). The Young’s modulus values reported for similar electrospun PLGA-based scaffolds were approximately in the range of 7–10 MPa (e.g., pure PLGA ≈ 7.10 ± 1.7 MPa and PLGA/collagen ≈ 9.8 ± 1.9 MPa) [27]. The elastic modulus of the mesh obtained in this study was within a comparable range, suggesting that the mesh may serve as mechanical support for the hydrogel layer. In addition, the porous structure of the mesh was thought to have enhanced hydrogel integration into fibrous mesh during preparation of the implantation construct.

Degradation studies of the hydrogel showed approximately 40% weight loss within the first day and 50% after 30 days (Figure 2e). Moreover, the swelling degree of the hydrogel was found to be 1534 ± 148%, indicating its high-water retention capacity, with a 15-fold increase in weight compared to its dry weight.

#### 3.1.2. Immunophenotypic Characterization of WJ-MSCs

**Figure 3:**
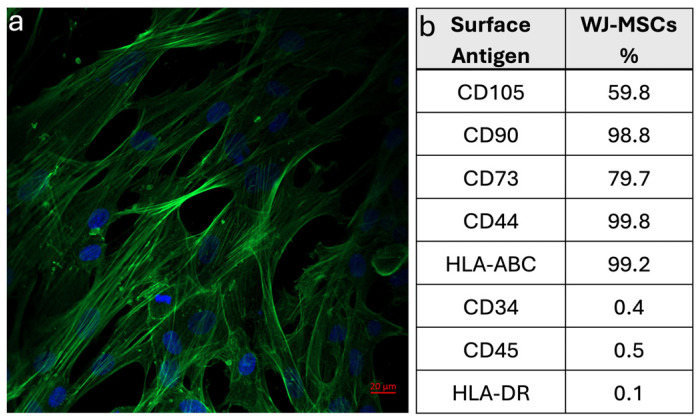
Morphological and immunophenotypic characterization of Wharton’s Jelly mesenchymal stem cells (WJ-MSCs). (**a**) LSCM image of WJ-MSCs stained with phalloidin-FITC (green) and DAPI (blue) for actin filaments and nuclei, respectively (magnification: 20×, scale bar: 20 µm). (**b**) Expression percentages (%) of cell surface antigens of WJ-MSCs determined by flow cytometry analysis.

WJ-MSCs adhered to fibronectin-coated surfaces and displayed fibroblastic morphology with well-organized cytoskeleton and nuclei, indicating typical healthy, adherent MSCs in culture (Figure 3a). Cell surface marker analysis of WJ-MSCs using flow cytometry demonstrated that cells isolated from the umbilical cord matrix exhibited positive expression of MSC markers CD105, CD90, CD44, CD73, and HLA-ABC, and negative expression of hematopoietic markers CD34, CD45, and HLA-DR (Figure 3b). These findings indicated that the cells were MSCs, exhibiting a surface antigen profile characteristic of MSCs.

#### 3.1.3. Viability of WJ-MSCs in the Hydrogel

**Figure 4:**
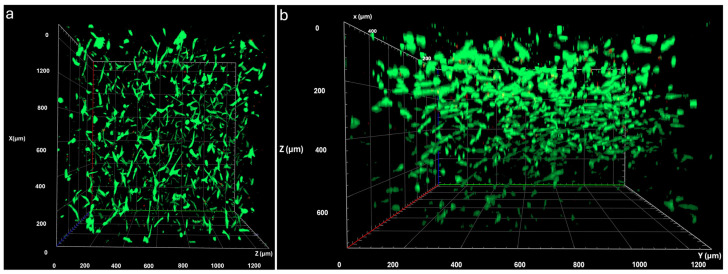
LSCM images of WJ-MSCs after a live/dead assay showing cell viability following 5 days of culture in the hydrogel. Alive cells are shown in green and dead cells in red. The three-dimensional images are presented as (**a**) top view and (**b**) side view to show the cell distribution within the 3D hydrogel.

For viability analysis, WJ-MSCs were loaded into the hydrogel and cultured for 5 days, and their viability was assessed using a live/dead assay. The results showed that WJ-MSCs exhibited high viability, with 92.2% of cells alive, and displayed cell–cell interactions within the hydrogel (Figure 4). 3D LSCM images showed that the cells exhibited spatial organization and were homogeneously distributed within the 3D hydrogel. Similarly, it has been shown that rat bone marrow stem cells (rBMSCs) can adhere, proliferate, and migrate to the interior of collagen type I hydrogels [28].

### 3.2. In Vivo Experiments

#### Immune Response Assessment

**Figure 5:**
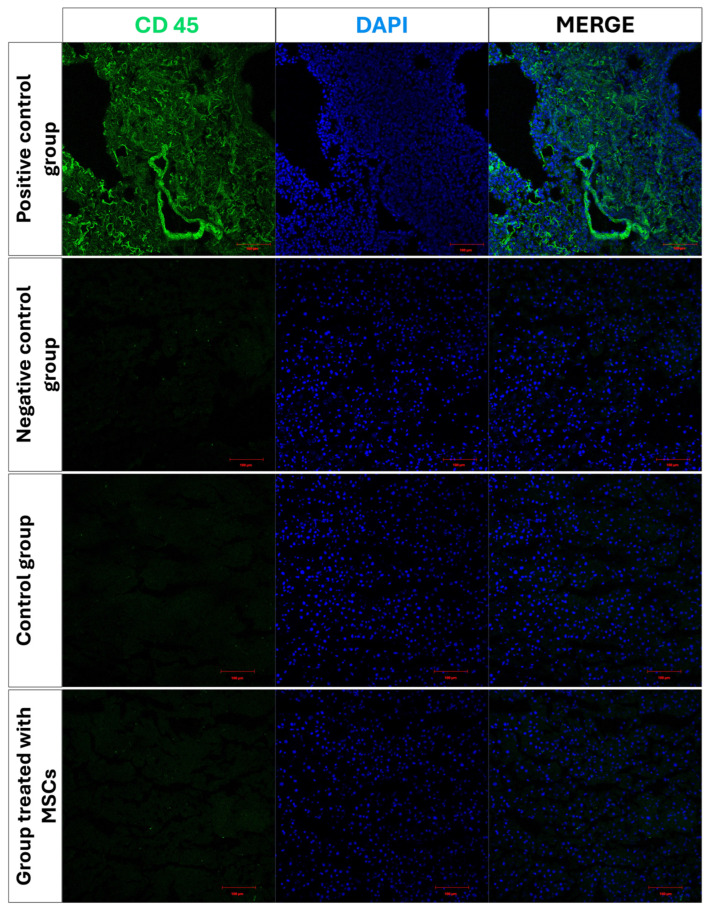
LSCM images of liver tissue samples after immunostaining against CD45 (green) and counterstained with DAPI (blue) for nuclei. Scale bar: 100 µm.

Immunostaining and LSCM analyses of liver tissues revealed no significant differences in CD45 expression between the control group and the WJ-MSC-treated group (*p* > 0.05). This result supports that the applied cell-loaded hydrogel system does not increase the immune response and is safe in terms of biocompatibility (Figure 5).

### 3.3. Biochemical Results

#### 3.3.1. Serum ALT, AST, ALP, and Total Bilirubin Levels Results

**Figure 6:**
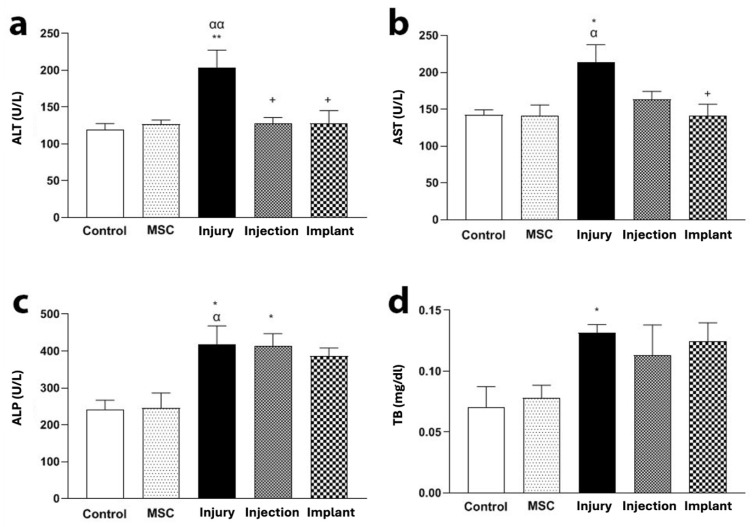
Serum levels of liver enzymes in experimental groups. (**a**) ALT serum levels were significantly increased in the injury group compared to the control and MSC groups (** *p* < 0.01, ^αα^ *p* < 0.01); ALT levels were significantly decreased in the injection and implant groups compared to the injury group (^+^ *p* < 0.05). (**b**) AST serum levels were increased in the injury group compared to the control and MSC groups (* *p* < 0.05, ^α^ *p* < 0.05); AST levels in the implant group were significantly lower compared to the injury group (^+^ *p* < 0.05). (**c**) ALP serum levels were increased in the injury and injection groups compared to the control group (* *p* < 0.05, ^α^ *p* < 0.05); no significant differences were found in ALP levels between the injury and injection groups or the injury and implant groups (*p* > 0.05). (**d**) TB serum levels were increased in the injury group compared to the control and MSC groups (* *p* < 0.05); no significant differences were observed in TB levels between the injury and injection groups or the injury and implant groups (*p* > 0.05).

The ALT level of the injury group was increased compared to the control and MSC groups (*p* < 0.01). In addition, the injection and implant groups presented low ALT levels compared to the TAA group (*p* < 0.05) (Figure 6a). The AST level of the injury group was increased compared to the control and MSC groups (*p* < 0.05). Furthermore, the AST level of the implant group was lower than the injury group (Figure 6b). The ALP levels of the injury and injection groups were higher than the control group (*p* < 0.05). There were no significant differences in the serum levels of ALP between the injury and injection groups or the injury and implant groups (*p* > 0.05) (Figure 6c). In addition, the TB level of the injury group was increased compared to the control and MSC groups (*p* < 0.05). However, no significant differences were noticed in the serum levels of TB between the injury and injection groups or the injury and implant groups (*p* > 0.05) (Figure 6d).

#### 3.3.2. MDA and GSH Levels and MPO and SOD Activity Results

**Figure 7:**
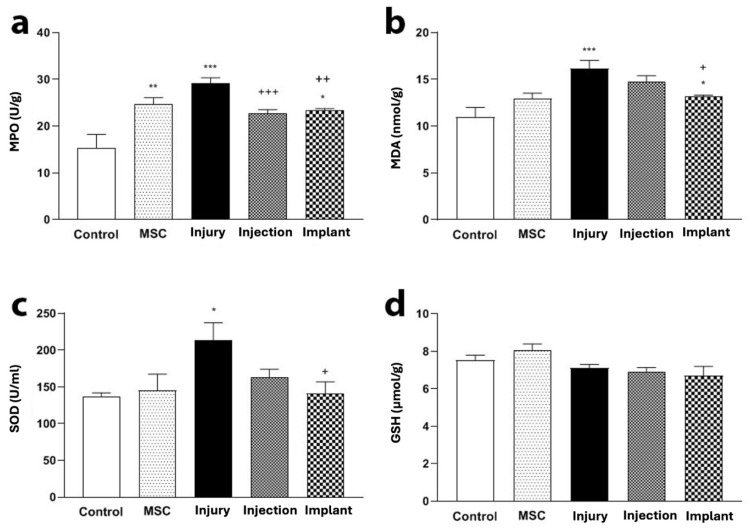
Serum levels of oxidative stress and antioxidant markers in different experimental groups. (**a**) MPO activity was significantly increased in the injury group compared to the control group (*** *p* < 0.001, ** *p* < 0.01, * *p* < 0.05), while it was significantly decreased in the injection and implant groups compared to the injury group (^+++^ *p* < 0.001, ^++^ *p* < 0.01). (**b**) MDA level was significantly higher in the injury group compared to the control group (*** *p* < 0.001, * *p* < 0.05), whereas it was significantly lower in the implant group compared to the injury group (^+^ *p* < 0.05). (**c**) SOD activity was significantly increased in the injury group compared to the control group (* *p* < 0.05), but it was lower in the implant group compared to the injury group (^+^ *p* < 0.05). (**d**) No significant differences were observed in serum GSH levels between the injury and injection groups or the injury and implant groups (*p* > 0.05).

MPO activity in the injury group was higher than the control group (*p* < 0.001), while this activity in the injection and implant groups was lower compared to the injury group (*p* < 0.05) (Figure 7a). MDA levels in the injury group were increased compared to the control group (*p* < 0.001), while in the implant group, they were lower than the injury group (*p* < 0.05) (Figure 7b). SOD activity in the injury group was increased compared to the control group (*p* < 0.05). The SOD activity in the implant group was lower than in the injury group (*p* < 0.05) (Figure 7c). No significant differences were observed in the serum GSH between the injury and injection groups or the injury and implant groups (*p* > 0.05) (Figure 7d).

### 3.4. Histopathological Results

**Figure 8:**
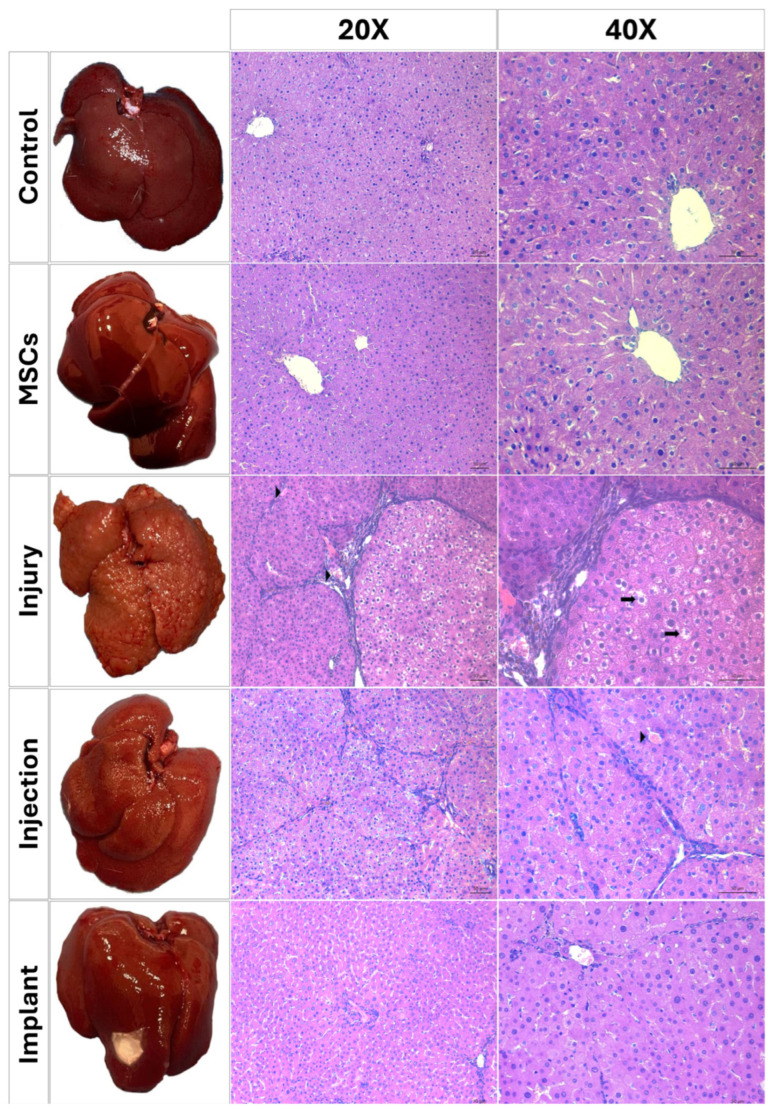
Light microscopic images of Hematoxylin and Eosin-stained liver tissue sections at different magnifications. Black arrows indicate vacuolization in hepatocytes, while black arrowheads indicate vasocongestion. Scale bars: at 10× magnifications, 100 µm; at 20× and 40× magnifications, 50 µm.

**Figure 9:**
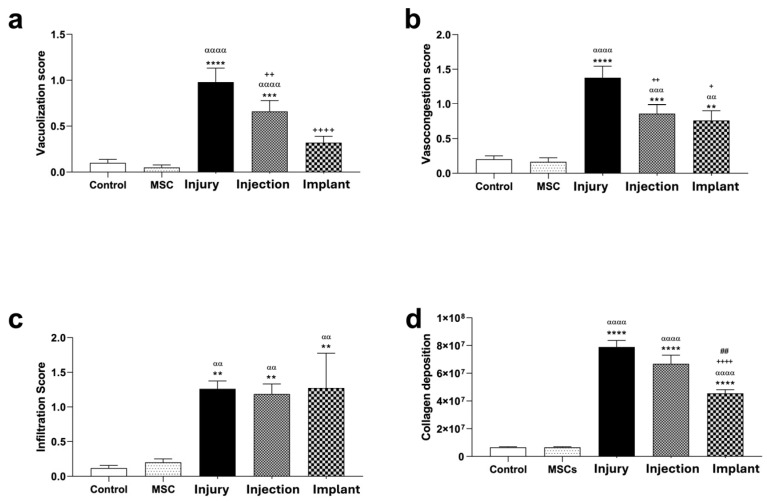
Quantitative analysis of histopathological parameters among experimental groups. (**a**) Vacuolization level was significantly increased in the injury group compared to the control group and MSC group (**** *p* < 0.0001, *** *p* < 0.001 and ^αααα^ *p* < 0.0001), while it was significantly reduced in the injection and implant groups compared to the injury group (^++++^ *p* < 0.0001, ^++^ *p* < 0.01). (**b**) Vasocongestion score was significantly higher in the injury group compared to the control and MSC group (**** *p* < 0.0001, *** *p* < 0.001, ** *p* < 0.01, ^αααα^ *p* < 0.0001, ^ααα^ *p* < 0.001 and ^αα^ *p* < 0.01), whereas it was significantly reduced in the injection and implant groups compared to the injury group (^++^ *p* < 0.01, ^+^ *p* < 0.05). (**c**) Infiltration score was significantly increased in the injury group compared to the control and MSC group (** *p* < 0.01, ^αα^ *p* < 0.01) and remained elevated in the injection and implant groups. (**d**) Collagen deposition was significantly increased in the injury group compared to the control and MSC group (**** *p* < 0.0001, ^αααα^ *p* < 0.0001), while it was significantly reduced in the implant group compared to the injury group (^++++^ *p* < 0.0001 vs. injury group; ^##^ *p* < 0.01 vs. injection group).

Light microscopic examination of H&E-stained liver sections from the control and MSC groups showed a normal parenchymal morphology. Hepatocytes with normal cytoplasmic morphology were radially arranged, extending from the central vein toward the sinusoids in each lobule (Figure 8). Vacuolization, infiltration, and vasocongestion, both in hepatocytes and the parenchyma, were histopathologically scored. No significant differences were found between the control and MSC groups in terms of these parameters (*p* > 0.05), indicating that the MSC application-only did not cause any significant tissue damage. However, TAA application significantly increased the levels of vacuolization, infiltration, and vasocongestion compared to the control and MSC groups (*p* < 0.01). Injection and implant treatments significantly reduced the levels of hepatocellular vacuolization compared to the injury group (*p* < 0.01). The degree of leukocytic infiltration in these groups remained significantly higher than in the control and MSC groups (*p* < 0.01). This suggests that these treatments had a limited effect on leukocytic infiltration while reducing the cytoplasmic vacuolization. In terms of vasocongestion, injection and implant treatments significantly reduced vasocongestion compared to the injury group (*p* < 0.05), demonstrating the potential therapeutic effects of these treatments. However, no significant differences were detected between the injection and implant groups in terms of any histopathological criteria (*p* > 0.05) (Figure 9).

**Figure 10:**
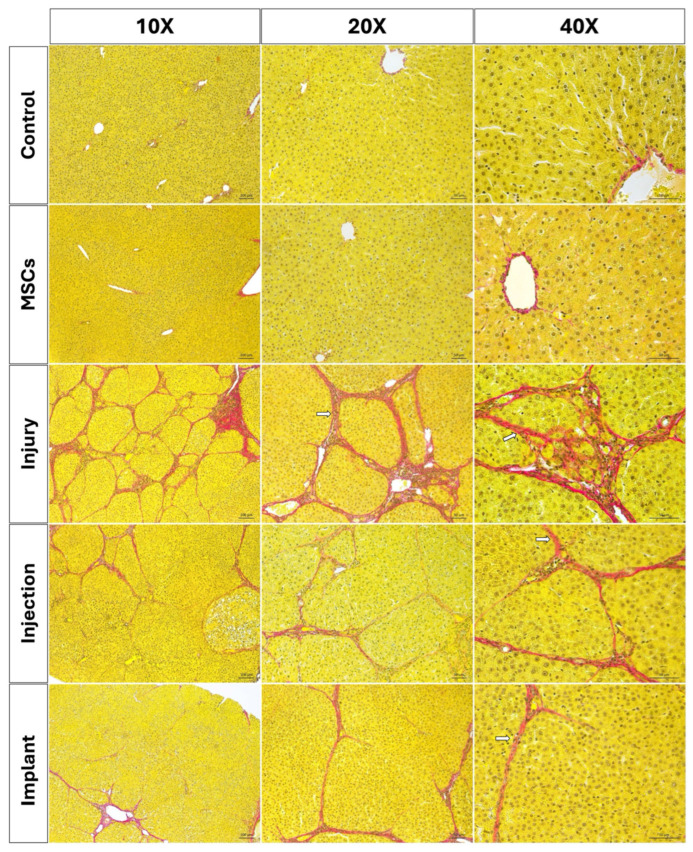
Light microscopic images of Sirius red-stained liver tissue sections. White arrows indicate fibrotic septa structures. Scale bars: at 10× magnifications, 100 µm; at 20× and 40× magnifications, 50 µm.

The Sirius red staining method was applied to paraffin-embedded sections to evaluate the distribution and structure of collagen fibers [29]. In the control and MSC groups, normal amount of collagen fibers was observed in the portal triad. Similarly, regular collagen fiber distribution was observed in the periportal area in the WJ-MSC-injected rats. Both the control group and the MSC-injected rats exhibited normal distribution of collagen fibers with regularly delineated lobular structures. However, a significant increase in collagen fibers was observed in the periportal region of liver sections from thioacetamide-injected rats (injury group). Sirius red staining indicated bridging fibrosis through the development of filamentous fibrosis as well as portal–portal and portal–central septa in the thioacetamide-injected rats (Figure 10). The degree of fibrosis was measured by Image J and statistically evaluated using GraphPad Prism 8.0 (GraphPad Software, San Diego, CA, USA). According to the results, no statistically significant differences were observed in terms of collagen accumulation between the injury group and the group in which the stem cell and melatonin-loaded hydrogel were intraperitoneally injected. However, in the group where the mesh, stem cell, and melatonin-loaded hydrogel combination was implanted, a significant decrease in collagen fibers was determined compared to the injury group (*p* < 0.01). Statistically significant differences were observed between the injury and implant, as well as the injection and implant groups. No significant differences were detected between the injury and injection groups (*p* > 0.05) (Figure 9d).

#### 3.4.1. Immunofluorescence Analysis

**Figure 11:**
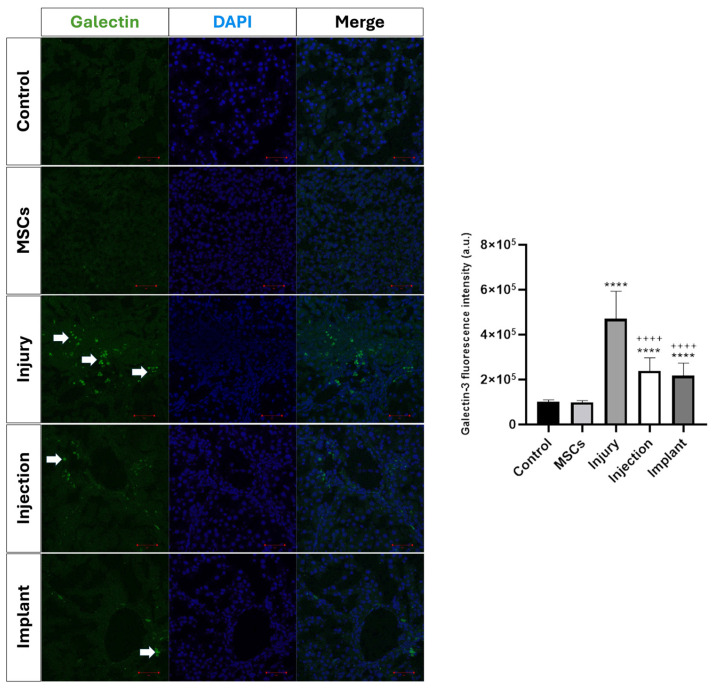
Quantitative analysis of galectin-3 immunofluorescence intensity and corresponding bar graph. Galectin-3 immunofluorescence signal intensity was measured using ImageJ, and data are presented as mean ± SD. Galectin-3 immunofluorescence intensity was significantly increased in the injury group compared with the control and MSCs groups (**** *p* < 0.0001). In the injection and implant groups, galectin-3 immunofluorescence intensity was significantly reduced compared with the injury group (^++++^ *p* < 0.0001), while no significant difference was observed between the injection and implant groups (*p* > 0.05). White arrows indicate galectin-3 expression. Scale bars: 50 µm.

Immunofluorescence staining was performed to evaluate the tissue-level distribution of galectin-3, which is known to play a critical role in regulating inflammation and extracellular matrix accumulation in liver fibrogenesis, and whose expression is associated with fibrosis severity [30]. Quantitative analysis of galectin-3 immunofluorescence intensity revealed a marked increase in the injury group compared with both the control and MSCs groups (*p* < 0.0001). No significant difference was observed between the control and MSCs groups (*p* > 0.05). Galectin-3 immunofluorescence intensity was significantly reduced in the injection and implant groups compared with the injury group (*p* < 0.0001). Both injection and implant groups also showed significantly lower galectin-3 immunofluorescence intensity compared with the control and MSCs groups (*p* < 0.0001). No significant difference was detected between the injection and implant groups (*p* > 0.05) (Figure 11).

#### 3.4.2. Transmission Electron Microscopy Results

**Figure 12:**
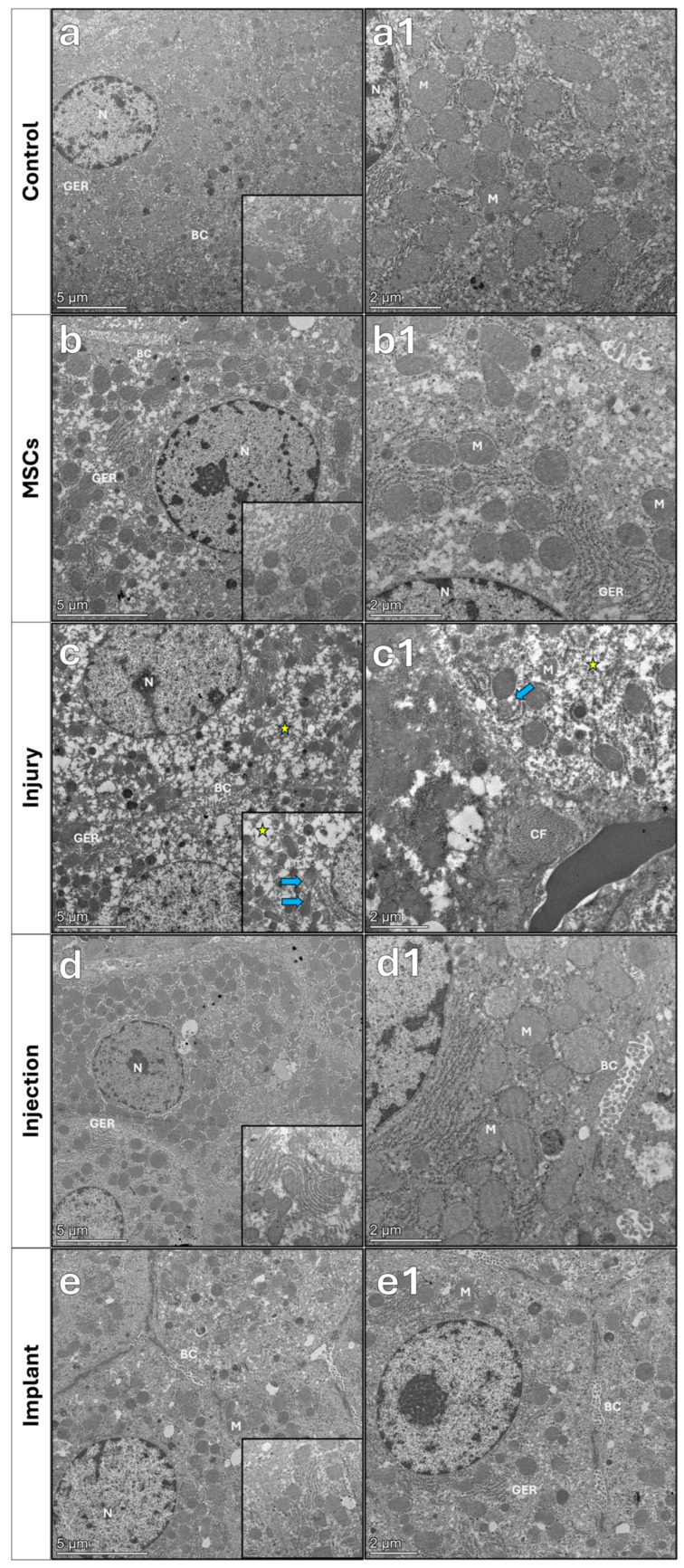
Normal ultrastructure was observed in the control and MSC groups, whereas the injury group showed hepatocellular damage characterized by dilated endoplasmic reticulum and irregular parenchymal organization. Treatment groups exhibited improved ultrastructural preservation. Insets show higher magnification of representative regions, granular endoplasmic reticulum (GER), and mitochondrial ultrastructure. Yellow stars indicate dilated membranes of endoplasmic reticulum, and blue arrows indicate mitochondria with disrupted ultrastructure. N: nucleus; BC: bile canaliculus; GER: granular endoplasmic reticulum; M: mitochondria; CF: collagen fibers. Scale bars: 5 µm (**a**–**e**), 2 µm (**a1**–**e1**), 1 µm (insets).

Transmission electron microscopy revealed normal ultrastructure of the liver parenchyma and hepatocytes in the control and MSC groups. Nuclei and nucleoli of the hepatocytes, as well as organelles, presented normal ultrastructures. Bile canaliculi with regular microvilli were observed on the lateral surfaces of hepatocytes. In the injury group, distinct damage was detected in the swollen hepatocytes. In particular, extensive vacuolization in hepatocytes, dilated membranes of endoplasmic reticulum (ER), and erased mitochondrial cristae were observed. Microvilli of bile canaliculi were distorted in the liver parenchyma. In the liver tissue sections of the implant group, nuclei of the hepatocytes maintained their ultrastructure close to normal. The parenchyma showed dilated ER membranes and a reduction in degenerative changes associated with vacuolization, while the bile canaliculi exhibited a fine structure close to normal (Figure 12).

## 4. Discussion

This study investigated the therapeutic effects of a collagen-based hydrogel containing WJ-MSCs and melatonin, either alone via injection or in combination with a fibrous mesh via implantation, to treat TAA-induced liver fibrosis. Based on the morphological results, the implantation construct composed of hydrogel and fibrous mesh was more effective in reducing fibrosis in liver tissue compared to the hydrogel injection application. This enhanced effect of the implant may be associated with improved local retention and stabilization of the therapeutic components at the tissue surface, preventing leakage of cells and melatonin. This result is consistent with the literature, highlighting the prominent role of biomaterial-assisted cellular therapies in regenerative medicine [21].

In experimental animals, TAA, which causes liver cirrhosis very similar to cirrhosis in humans, can be administered orally or intraperitoneally and causes fibrosis and cirrhosis depending on the dose and duration of exposure. It has been reported that cirrhosis effectively occurs in 2–3 months with intraperitoneal administration [7,8,9]. In our study, TAA was administered to rats at 200 mg/kg three times a week for 6 weeks, and fibrosis was induced in the animals. The TAA-induced liver fibrosis model was histopathologically characterized by ballooning degeneration of hepatocytes, vasocongestion of sinusoids, and leukocyte infiltration in portal areas in the injury group. Similar findings have been reported in previous studies demonstrating the hepatotoxic effects of TAA leading to fibrosis [31,32]. In the present study, histochemical analysis using Sirius red staining revealed a significant increase in collagen fiber accumulation in the group treated with TAA alone. These findings are consistent with previous studies, demonstrating the hepatotoxic effects of TAA to trigger a fibrotic response [33,34,35]. In addition, our findings are consistent with previous studies supporting the role of galectin-3 in fibrogenic and inflammatory processes in chronic tissue injury. In experimental hepatic fibrosis models, galectin-3 expression is upregulated in fibrotic liver tissue and is temporally and spatially associated with extracellular matrix deposition. Genetic disruption or knockdown of galectin-3 markedly attenuates myofibroblast activation and fibrosis in vivo, indicating its critical contribution to fibrogenesis and tissue remodeling after liver injury [36].

Moreover, in a cirrhosis model, increased galectin-3 expression was closely linked to fibrosis development, and therapeutic interventions that reduced galectin-3 levels were associated with decreased inflammatory cell infiltration and fibrotic areas [37].

There are studies demonstrating the ameliorating effects of melatonin, a well-known antioxidant, on liver fibrosis [2,14,38,39]. In a study evaluating the effects of melatonin administration on healing in a liver fibrosis model, mice received 200 mg/kg of TAA intraperitoneally twice a week. Sirius red staining revealed increased collagen deposition following TAA administration. Oral melatonin administered at 10 mg/kg/day as a pretreatment, initiated before TAA administration, was shown to significantly prevent TAA-induced liver damage [40].

Umbilical cord matrix-derived WJ-MSCs are an appropriate cell source in regenerative medicine studies due to their high proliferation and differentiation capacities, suitability for xenogenic applications, and hypoimmunogenicity [12,13]. In a study investigating TAA-induced liver fibrosis in mice, TAA was administered intraperitoneally to mice twice weekly for 6 weeks to induce fibrosis. Human WJ-MSCs were then administered as a single infusion via the tail vein, and their effects were evaluated for 2 weeks. WJ-MSC treatment reduced collagen deposition and was reported to regress liver fibrosis [41]. In some studies, WJ-MSCs were combined with melatonin and showed a synergistic effect on liver fibrosis. In another study where liver fibrosis was induced using CCl_4_, the effects of melatonin and MSCs, both individually and in combination, were evaluated. Morphological and histopathological results, as well as biochemically analyzed bilirubin and ALT levels, showed a significant reduction compared to other groups. The results indicate that the combined administration of MSCs and melatonin demonstrates a strong therapeutic effect in CCl_4_-induced liver fibrosis compared to MSCs and melatonin alone [11]. In our study, intending to increase its effectiveness, we also combined the melatonin with WJ-MSCs for the treatment of liver fibrosis. The histological, ultrastructural, and biochemical results were consistent with the literature [11,16,42].

Polymeric biomaterials in the form of hydrogels and fibrous meshes are widely used in tissue engineering and drug delivery, either alone or in combination [17,18,19]. They are particularly applied for the treatment of soft tissues due to their biocompatibility, biodegradability, and mechanical supportive properties. Biomaterials used in the treatment of liver fibrosis can act as a structural support and help deliver therapeutic agents in a more localized manner. In one study, encapsulation of mesenchymal stem cell-derived extracellular vesicles (EVs) in biodegradable polyethylene glycol (PEG) hydrogel resulted in a controlled release, significantly increased the antifibrotic, anti-inflammatory, antiapoptotic, and regenerative effects compared to single-dose EV administration. This improvement was confirmed by a reduction in fibrotic area, decreased α-SMA expression, and reduced serum ALT levels. These findings suggest that biomaterial-assisted systems can enhance therapeutic effects in liver fibrosis and highlight their importance in its treatment [43].

Although no specific studies on melatonin and WJ-MSCs-loaded collagen-based hydrogel systems directly used for liver regeneration have been found in the literature, studies have shown that melatonin-containing biopolymeric scaffolds could provide early activation of antioxidant and regenerative effects [44]. There have been studies in which either stem cells or melatonin have been combined with fibrous scaffolds or hydrogels [18,19,21]. In a study using a 200 mg/kg dose of TAA for two consecutive days, acute liver failure was induced in a liver injury model. MSCs loaded onto a PLGA scaffold were shown to increase survival and function in this model, and their paracrine effects were enhanced. Furthermore, it has been reported that MSCs retained on the scaffold can partially differentiate into hepatocyte-like cells, even without migrating into liver tissue. These findings, as observed in our study, support the fact that biomaterial-supported cellular applications significantly enhance the regenerative response in TAA-induced liver injury [45]. However, our literature search did not reveal any construct containing both stem cell and melatonin-loaded hydrogel integrated with an electrospun mesh.

In the present study, the therapeutic effects of a collagen-based hydrogel containing WJ-MSCs and melatonin were investigated by comparing injection and implantation applications in TAA-induced liver fibrosis using histopathological, biochemical, and ultrastructural analyses. Light microscopic examination of the control and MSC groups showed normal parenchymal morphology. TAA application significantly increased vacuolization, infiltration, and vasocongestion levels compared to the control and MSC groups. While injection and implant treatments significantly reduced hepatocellular vacuolization and vasocongestion levels compared to the injury group, the degree of leukocyte infiltration in these groups remained significantly higher than in the control and MSC groups. This suggests that these treatments had a limited effect on leukocytic infiltration while reducing cytoplasmic vacuolization. Since leukocyte infiltration is an indicator of inflammation, these findings suggest a limited anti-inflammatory effect. Although melatonin is known for its anti-inflammatory effects, no obvious anti-inflammatory response was observed in this study. This may be due to the selected dosages of melatonin and thioacetamide, the complexity of inflammatory processes in liver fibrosis, and the possibility that the treatment period was not sufficiently long to fully capture potential therapeutic effects of melatonin, which might become more evident with a longer follow-up. Previous studies have demonstrated that significant suppression of inflammatory mediators, such as NF-κB and iNOS, requires prolonged administration periods or higher cumulative doses, particularly in chronic liver injury models such as CCl_4_-induced fibrosis. [46,47]. Histopathological results demonstrated that Sirius red staining revealed prominent fibrotic septa formation in the periportal region in the injury group. This fibrotic response was partially attenuated in the injection group, while a significant decrease in collagen deposition was observed in the implant group. This suggests that implant application, which provides localized and targeted therapy, may be more effective than injection. In the present study, we observed a significant increase in galectin-3 immunofluorescence intensity in the injury group compared with the control and MSCs groups. Both injection and implant applications significantly reduced galectin-3 immunofluorescence relative to the injury group, suggesting that these treatments effectively attenuated injury-induced up-regulation of galectin-3. No significant differences were observed between the injection and implant groups.

Ultrastructural results showed that hepatocytes in the control and MSC groups exhibited normal nuclear morphology, dense cristae of mitochondria, and regular granular endoplasmic reticulum (GER) structures. In contrast, severe degenerative changes were observed in the injury group, including effacement of mitochondrial cristae, dilation of GER membranes, and erased microvilli in bile canaliculi [48]. This damage was partially reduced in the injection group, while cellular integrity was largely preserved in the implant group. These findings suggest that the implant application promotes regeneration at the cellular level and preserves hepatocyte function. In a study evaluating the effects of vitamin C and vitamin E on TAA-induced liver fibrosis, the ultrastructures of liver tissue samples were examined using transmission electron microscopy. The results revealed that vitamin C and vitamin E had a therapeutic effect on TAA-induced liver damage, such as endoplasmic reticulum fragmentation and mitochondrial damage [49]. These findings are consistent with the findings obtained with stem cell and melatonin therapy in our study.

Biochemical analyses revealed significant increases in ALT, AST, ALP, and total bilirubin levels in the injury group. These parameters are considered biochemical indicators of hepatocellular damage. Significant decreases in ALT and AST levels were observed in the injection and implant groups, with AST levels particularly decreasing to near-normal levels in the implant group. No significant differences were observed in ALP and total bilirubin levels among the treatment groups. This suggests that treatment is effective in reducing hepatocellular damage.

As oxidative stress parameters, a significant increase in MPO and MDA levels and a compensatory increase in SOD activity were observed in the injury group. These findings suggest that TAA exacerbates liver damage through oxidative stress. A significant decrease in MPO and MDA levels, as well as a decrease in SOD activity were observed in the implant group. This suggests that the treatment reduces the oxidative stress load and balances the antioxidant defense system. No significant differences were observed in GSH levels between the groups, suggesting that GSH metabolism may be affected over a longer period or at different doses. The biochemical findings reported in [49] support our current results. In a study using a TAA-induced liver fibrosis model (100 mg/kg BW, IP, every other day), vitamin D3 (VD3, 1000 IU/kg BW, IM, daily) treatment significantly reduced liver damage and fibrosis. VD3 administration decreased ALT, AST, ALP, and total bilirubin levels, while the oxidative stress marker MDA decreased as SOD and GSH increased [50].

While most biochemical, oxidative stress, and inflammatory parameters did not show significant differences between the injection and implant groups, a significant decrease in collagen deposition was observed in the implant group compared to the injection group, indicating an enhanced antifibrotic effect of the combined construct structure. In summary, these findings suggest that the application of a hydrogel containing WJ-MSCs and melatonin, combined with a fibrous mesh, improved histological and ultrastructural parameters of tissue regeneration in liver fibrosis. The observed therapeutic effect is likely mediated through the complementary actions of MSC-derived paracrine signaling and melatonin-induced reduction in oxidative stress, which together create a more favorable microenvironment for tissue regeneration [46,47]. It is possible that, in the injection group, a proportion of MSCs and melatonin may not have been fully retained at the target site prior to hydrogel gelation, which could have resulted in limited localization and reduced local therapeutic efficiency. In contrast, in the local implant application, fibrous mesh offers a more stable platform that supports hydrogel adhesion to the tissue surface, thereby enabling a more effective delivery of the cells and melatonin and providing enhanced anti-fibrotic effect.

Although the implantation approach demonstrated a more pronounced therapeutic effect, the injection approach also showed a marked improvement in liver regeneration parameters. The implantation method may offer the advantage of sustained localized delivery through enhanced retention at the tissue surface, whereas the injection approach provides a minimally invasive alternative with relevant translational potential, particularly for patients in whom surgical procedures may present a higher risk. Taken together, these results indicate that the choice of application method may be preferred according to disease severity and patient condition, rather than assuming a single universally superior approach.

The present study demonstrates that the combined application of WJ-MSCs, melatonin, collagen hydrogel, and a mesh-based system exerts significant therapeutic effects in a TAA-induced liver fibrosis model. Nevertheless, several limitations should be considered when interpreting these findings. The current design primarily reflects the overall efficacy of the combined system, while the specific contributions of individual components remain to be further elucidated. In addition, the incorporation of quantitative image analysis may further enhance statistical robustness. Future investigations integrating molecular pathway analyses and advanced tracking approaches would provide a more comprehensive understanding of the underlying mechanisms.

In conclusion, this study provides important preclinical data that demonstrate the combination of MSCs, antioxidants, and biomaterials may be an effective treatment approach in liver fibrosis. This implantation construct is believed to have significant translational potential for targeted and therapeutic strategies in the clinic while maintaining compatibility with biological systems.

## Data Availability

All data generated or analyzed during this study are included in this published article. Further inquiries can be directed to the corresponding author.

## Abbreviations

The following abbreviations are used in this manuscript:

ESLD	End-Stage Liver Disease
CCl_4_	Carbon Tetrachloride
TAA	Thioacetamide
HSCs	Hepatic Stellate Cells
MSCs	Mesenchymal Stem Cells
WJ-MSCs	Wharton’s Jelly Mesenchymal Stem Cells
MHC	Major Histocompatibility Complex
ROS	Reactive Oxygen Species
PGA	Polyglycolic Acid
PLA	Polylactic Acid
PLGA	Poly (D, L-Lactide Co-Glycolide)
Chl	Chloroform
DMF	N,N-Dimethylformamide
DMEM	Dulbecco’s Modified Eagle Medium
NaOH	Sodium Hydroxide
NaHCO_3_	Sodium Bicarbonate
HG	Hydrogel
Mel	Melatonin
SEM	Scanning Electron Microscopy
PBS	Phosphate-Buffered Saline
FBS	Fetal Bovine Serum
bFGF	Basic Fibroblast Growth Factor
Try-EDTA	Trypsin-Ethylenediaminetetraacetic Acid
CD	Cluster of Differentiation
FITC	Fluorescein Isothiocyanate
PerCP	Peridinin Chlorophyll Protein
HLA-ABC	Human Leukocyte Antigen—A, B, C
HLA-DR	Human Leukocyte Antigen—DR
LSCM	Laser Scanning Confocal Microscopy
ALT	Alanine Aminotransferase
AST	Aspartate Aminotransferase
ALP	Alkaline Phosphatase
TB	Total Bilirubin
SOD	Superoxide Dismutase
MPO	Myeloperoxidase
MDA	Malondialdehyde
HETAB	Hexadecyltrimethylammonium Bromide
K_2_HPO_4_	Dipotassium Hydrogen Phosphate
GSH	Reduced Glutathione
H&E	Hematoxylin and Eosin
DAPI	4′-6-Diamidino-2-Phenylindole
TEM	Transmission Electron Microscope
rBMSCs	Rat Bone Marrow Stem Cells
